# Unscrambling the Role of Redox-Active Biometals in Dopaminergic Neuronal Death and Promising Metal Chelation-Based Therapy for Parkinson’s Disease

**DOI:** 10.3390/ijms24021256

**Published:** 2023-01-09

**Authors:** Alfredo Gonzalez-Alcocer, Ana Patricia Duarte-Jurado, Adolfo Soto-Dominguez, Maria de Jesus Loera-Arias, Eliud Enrique Villarreal-Silva, Odila Saucedo-Cardenas, Roberto Montes de Oca-Luna, Aracely Garcia-Garcia, Humberto Rodriguez-Rocha

**Affiliations:** 1 Departamento de Histologia, Facultad de Medicina, Universidad Autonoma de Nuevo Leon, Francisco I. Madero S/N, Mitras Centro, Monterrey 64460, Mexico; 2 Servicio de Neurocirugía y Terapia Endovascular Neurológica, Hospital Universitario, Dr. Jose Eleuterio Gonzalez, Monterrey 64460, Mexico

**Keywords:** redox-active, biometals, iron, copper, chelation, Parkinson’s disease

## Abstract

Biometals are all metal ions that are essential for all living organisms. About 40% of all enzymes with known structures require biometals to function correctly. The main target of damage by biometals is the central nervous system (CNS). Biometal dysregulation (metal deficiency or overload) is related to pathological processes. Chronic occupational and environmental exposure to biometals, including iron and copper, is related to an increased risk of developing Parkinson’s disease (PD). Indeed, biometals have been shown to induce a dopaminergic neuronal loss in the substantia nigra. Although the etiology of PD is still unknown, oxidative stress dysregulation, mitochondrial dysfunction, and inhibition of both the ubiquitin–proteasome system (UPS) and autophagy are related to dopaminergic neuronal death. Herein, we addressed the involvement of redox-active biometals, iron, and copper, as oxidative stress and neuronal death inducers, as well as the current metal chelation-based therapy in PD.

## 1. Introduction

**Figure 1:**
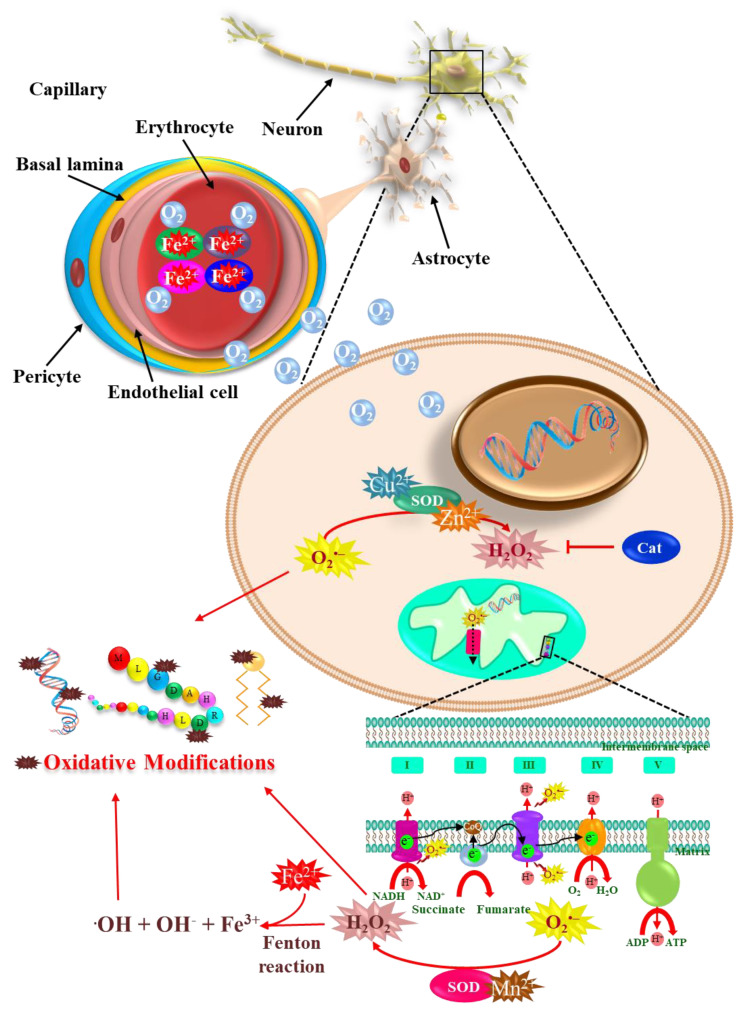
Biometals are essential for the proper function and maintenance of neuronal cells. Iron is mainly found in the erythrocytes, bound to hemoglobin, and is essential for oxygen transfer to all tissues, including neurons. Iron also takes place in the Fenton reaction producing free radicals. Zinc and copper, and manganese are cofactors required for the antioxidant enzymes SOD1 and SOD2, respectively. These enzymes are scavengers of superoxide anion, and their activity is critical in regulating oxidative stress in the cytoplasm and the mitochondria. Oxidative stress may ultimately induce DNA damage, protein oxidation, and lipid peroxidation with deleterious consequences.

Metals play essential biological functions in all living organisms. The human body requires minimal amounts of metallic elements to maintain a healthy development of physiological functions. However, metal levels outside the normal range may lead to the development of pathologies. An organism’s deficiency or excess of essential metals leads to severe biological alterations. However, not all metals are essential or beneficial to organisms; some nonessential and potentially toxic metals might be capable of causing undesirable effects on the genome, glycolysis, Krebs cycle, oxidative phosphorylation, and carbohydrate, lipid, protein, and amino acid metabolism [1]. Only biometals, including iron, copper, zinc, manganese, molybdenum, sodium, potassium, calcium, chromium, and cobalt, are indispensable for life. About 40% of all enzymes with known structures require biometals to function correctly [2]. These biometals are not limited to enzymatic activities but also play structural, electrostatic, energetic, and transport functions. Biometals directly affect by targeting biomolecules (DNA, lipids, proteins) (Figure 1), organelles, cells, tissues, organs, and the biological micro- and macro-environment, as they become part of it [3,4]. Living cells possess a redox metabolism where oxidation-reduction (redox) reactions occur in fundamental processes of redox regulation, collectively termed “redox signaling” and “redox control” [5]. Several studies performed on biological systems have shown that redox-active metals, including iron, copper, cobalt, chromium, and manganese, can undergo redox cycling reactions and produce reactive free radicals, also termed reactive oxygen species (ROS) or reactive nitrogen species (RNS) by upregulating Haber–Weiss and Fenton reactions and generating damage to cells [6].

The brain contains some of the highest iron, copper, zinc, and manganese concentrations in the human body [7]. These metals participate in synaptic transmission, myelinogenesis, energy production, and regulation of oxidative stress. Many biochemical processes rely on metals to transfer electrons via redox chemistry, neuronal excitation, protein structure, and enzymatic function [8].

The average human young brain consumes 20% of the oxygen taken in through respiration [9]. Because of high oxygen demand and cell complexity, high metal levels diffuse to the central nervous system (CNS) [10]. Therefore, the CNS is susceptible to metal damage [11]. Alterations in Fe, Cu, Zn, and Mn levels and distribution are associated with Parkinson’s Disease (PD) [12].

This review discusses the role of biologically essential redox-active biometals, iron, and copper, as oxidative stress and neuronal death inducers and the current metal chelation-based therapy in PD.

## 2. Metals, Parkinson’s Disease, and Oxidative Stress

Humans are regularly exposed to electromagnetic radiation, pollutants, and cellular metabolism byproducts that generate free radicals. Free radicals have an unpaired electron in their outer orbit [13,14], and oxygen radicals are involved in many cellular biochemical activities, such as signal transduction and gene transcription [15]. The most common cellular free radicals are hydroxyl (OH^•^), superoxide anion (O_2_^•–^), and nitric monoxide (NO^•^). Even some other species, such as hydrogen peroxide (H_2_O_2_) and peroxynitrite (ONOO^–^), are highly reactive but are not free radicals; however, they can generate free radicals [16]. Mammalian cells produce free radicals and ROS as byproducts through physiological and biochemical processes, primarily due to aerobic metabolism [17]. Because of this, cells have an effective antioxidant defense involving glutathione, arginine, vitamins E, C, and A, and antioxidant enzymes to regulate ROS generation [18].

Overproduction of reactive species can cause oxidative damage to biomolecules (lipids, proteins, DNA), leading to chronic diseases such as atherosclerosis, cancer, diabetes, rheumatoid arthritis, myocardial infarction, chronic inflammation, and cardiovascular and neurodegenerative diseases in humans [19]. 

Oxidative and nitrative stress in the mesencephalon, where dopaminergic neurons are located, is one of the main factors related to PD pathogenesis [20,21]. Dopamine is susceptible to auto-oxidization, producing toxic semiquinone species, H_2_O_2_, and a small amount of the neurotoxin 6-hydroxydopamine [22,23]. A study performed in post-mortem idiopathic PD brains and neurologically healthy adult brains matched by age showed that glutathione peroxidase activity was slightly but significantly reduced in several brain areas, including substantia nigra in PD brain samples [24]. A decrease in mitochondrial complex I activity has been reported in the substantia nigra of PD patients, which ultimately increases oxidative stress [25].

Chronic occupational and environmental exposure to metals, including iron and copper, increases the risk of developing PD [26]. Abnormally high iron and zinc levels have been detected in the substantia nigra of PD patients’ post-mortem samples. A deficiency or an overload of metals may influence the appearance of this disorder [27]. Metal ions and byproducts of the electron transport chain play a crucial role in forming intracellular free radicals leading to oxidative stress, where the imbalance of free radicals, antioxidants, and detoxifying enzymes occurs [28]. As a result, oxidatively modified molecules such as nucleotides, proteins, and lipids accumulate in the cellular compartment provoking dysfunction [29]. Therefore, the lack of control of the defense system, especially in sensitive cells such as neurons, eventually will lead to cell death [28,30].

**Figure 2:**
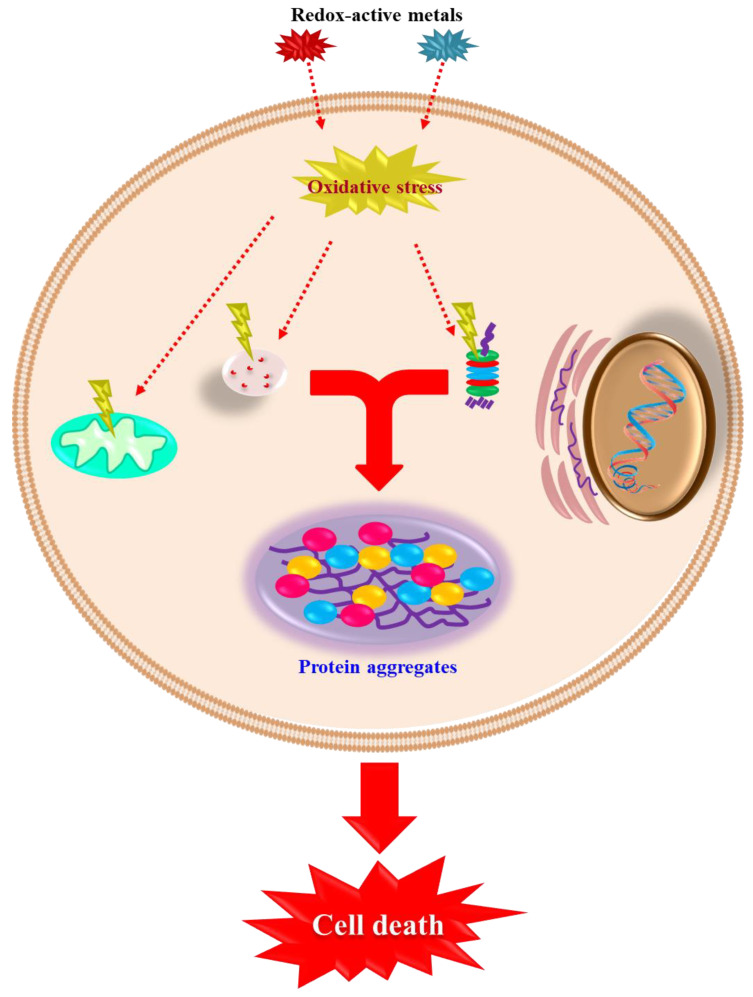
Interrelated events involved in the neurodegeneration process. Redox-active metals, including iron and copper, are recognized as inducers of oxidative stress. The latter relates to mitochondria dysfunction, lysosomal, and proteasomal degradation pathways inhibition, and ulterior protein accumulation and aggregation. Both protein degradation pathways may compensate for each other’s disruption and are affected in Parkinson’s Disease (PD) patients, where a more advanced state is characterized by Lewy body appearance and dopaminergic neuronal loss.

Lewy bodies (LB) are abnormal protein deposits containing α-synuclein associated with ubiquitin and tau, among other proteins (Figure 2). Multivalent metal ions such as iron, copper, and manganese increase α-synuclein fibril formation by inducing conformational changes [31,32,33]. Oxidative modifications and phosphorylation may engage both protein activity and half-life. Phosphorylated proteins strongly bind to certain metals [34,35,36].

Although non-enzymatic antioxidants have shown neuroprotective effects in PD experimental models, they have failed to reproduce this protection in clinical trials [37]. Therefore, it is imperative to understand the mechanisms involved in PD to explore diverse potential therapeutics more efficiently.

## 3. How Cells Die: Classical Mechanisms of Cell Death

Before discussing the role of biometals in neuronal death, it is worth summarizing the classical mechanisms of cell death. According to morphological, biochemical, and genetic characteristics, cell death is classified into three major types: apoptosis, necrosis, and autophagy. However, another section will discuss the latter, and additional cell death mechanisms will briefly be described. 

Apoptosis is well-characterized and known as programmed cell death type I (PCD type I). Its morphological changes include cell shrinkage, chromatin condensation (pyknosis), nuclear fragmentation (karyorrhexis), loss of plasma membrane integrity, and plasma membrane blebbing forming apoptotic bodies [38]. 

Necrosis, usually described as an accidental and uncontrollable mechanism, shows a substantial gain in cell volume (oncosis), swollen organelles, and disruption of the plasma membrane with the subsequent intracellular content release [39]. However, according to recent findings and following the guidelines of the Nomenclature Committee on Cell Death 2018, this classification has been updated, and mitochondrial permeability transition (MPT)-driven necrosis and necroptosis are now included, among other cell death subroutines [38]. MPT-driven necrosis is a regulated cell death induced by cell microenvironment disturbances that alter the inner mitochondrial membrane impermeability and is cyclophilin D (CYPD)-dependent [40,41,42]. Necroptosis is a regulated process activated by the recognition of extracellular and intracellular triggers through death receptors, and mixed lineage kinase domain-like pseudokinase (MLKL), receptor-interacting protein kinase 1 (RIPK1), and RIPK3 are crucial signaling molecules [43]. Ferroptosis is an intracellular iron- and ROS-dependent cell death mechanism that involves strong lipid peroxidation, glutathione peroxidase-4 depletion, glutathione imbalance, and mitochondria morphological alterations, including increased mitochondria membrane electron density, decreased or loss of cristae, and outer mitochondrial membrane rupture [44]. 

## 4. Redox-Active Metals’ Role in Dopaminergic Neuronal Death

### 4.1. Iron

Iron is the most abundant metal on Earth, and almost all organisms have evolved to use this ubiquitous transition metal [45]. This metal is essential for the human body’s proper functioning. Iron is vital for oxygen transport (bound to hemoglobin), oxidative phosphorylation (bound to cytochrome C), neurotransmitter synthesis, myelin formation, and regulation of the biosynthesis of proteins such as ferritin and transferrin receptor (to store or mobilize iron) through iron-response proteins binding to iron-responsive elements at mRNA level [46,47,48]. 

**Figure 3:**
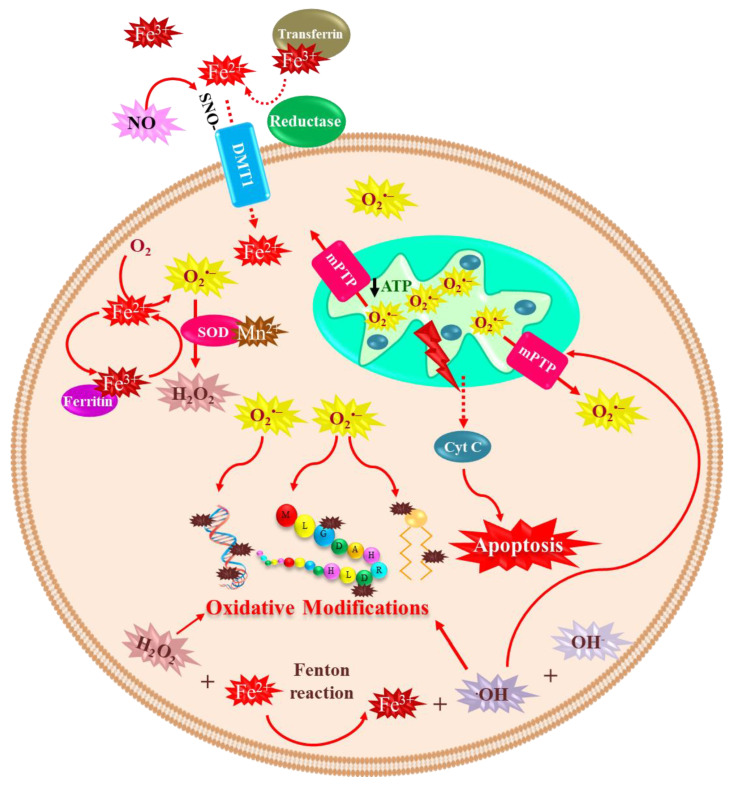
Iron toxicity in neuronal cells is mediated by oxidative stress. Fe^3+^ is reduced to Fe^2+^ to enter the cell, and DMT1 is the major iron importer in neurons. Iron toxicity is induced by a sequence of highly oxidative and toxic reactions as it takes place in the Fenton reaction producing free radicals. Fe^2+^ reacts with H_2_O_2_ or lipid peroxides to generate Fe^3+^, OH^−^, and hydroxyl radical (OH^•^) or lipid radicals, which may lead to oxidative damage of macromolecules. Accumulation of OH^•^ leads to activation of the mitochondrial permeability transition pore (mPTP), which temporarily opens and increases reactive oxygen species (ROS). It provokes apoptotic cell death triggered by decreased ATP production, mitochondrial swelling, and rupture of the outer mitochondrial membrane, with subsequent release of cytochrome C to the cytosolic compartment.

In contrast, unbound iron causes cell toxicity as it can trigger a series of highly oxidative and toxic reactions [49]; this occurs when the iron concentrations exceed the binding capacity of transferrin [50]. Iron has a wide range of oxidative states, Fe^2+^ (ferrous) and Fe^3+^ (ferric) being the most common in biological environments [51] (Figure 3). Fe^2+^ binding to proteins is very unstable [52]. Iron’s neurotoxic effect has been related to the divalent metal ion transporter 1 (DMT1) overexpression, which imports iron into the cell, and can also be inhibited by H-ferritin [53]. Moreover, S-nitrosylation (SNO) of DMT1 cysteine thiol enhances Mn^2+^ and Fe^2+^ uptake [54]. Additionally, SNO-DMT1 has been detected in the post-mortem substantia nigra of PD patients [54]. The redox state of iron determines its role in cytotoxic reactions [55].

In a healthy brain, iron is distributed in a specific pattern by region and cell type. It is abundant in the substantia nigra and the basal ganglia, which are rich in dopaminergic neurons [56]. 

For over four decades, changes in iron and ferritin levels have been described in the brain of PD patients [27]. Several mechanisms are associated with iron-induced dopaminergic cell death, including (1) Fenton redox-reactions producing hydroxyl radicals [57]; (2) DA oxidative deamination, which is catalyzed by monoamine oxidase B (MAO-B) and regulated by Fe^2+^ and Fe^3+^ [58,59]; (3) 6-hydroxydopamine neurotoxic formation through DA metabolites reaction with iron and H_2_O_2_ [60]; and (4) increased rate of iron-induced α-synuclein fibril formation [33]. Iron takes part in the Fenton reaction producing free radicals; Fe^2+^ reacts with H_2_O_2_ or lipid peroxides to generate Fe^3+^, hydroxyl ion (OH^–^), and OH^•^ or lipid radicals, which may lead to oxidative damage of macromolecules [47,61]. The Haber–Weiss reaction is where hydroxyl ion and hydroxyl radical are generated from the reaction of H_2_O_2_ and O_2_^•–^ catalyzed by iron [62]. Additionally, the formation and accumulation of OH^•^ lead to activation of the mitochondrial permeability transition pore (mPTP), which temporarily opens and increases ROS, provoking long-lasting activation and cell death. The latter is triggered by a decrease of ATP production, mitochondrial swelling, and rupture of the outer mitochondrial membrane, with subsequent release of mitochondrial death factors such as cytochrome C to the cytosolic compartment activating cell death by apoptosis [63]. Moreover, p53 is involved in mitochondrial dysfunction and oxidative stress mediated by Fe^2+^ in neuronal synaptic terminals [64].

Interestingly, lysosomes contain a redox-active iron pool derived from iron-rich macromolecules and cellular organelles, such as ferritin and mitochondria [65,66]. Most iron is found in a non-redox active form bound to ferritin. Ferritin degradation inside lysosomes during autophagy may be an intracellular redox-active iron source [67]. Next, H_2_O_2_ diffuses into lysosomes and reacts with the iron species through the Fenton and Fenton-like reactions, resulting in hydroxyl radical generation [65]. Intriguingly, defective mitochondria and lysosomes may promote RIPK1 activation, making cells susceptible to necroptosis [68]. Recently, necroptosis’s partial contribution to iron-mediated toxicity was demonstrated by using iron chelator deferoxamine (DFO) and the necroptosis inhibitor necrostatin 1 (NEC-1), significantly reducing cell death rates in the glutamate-induced model in vitro [69].

Iron accumulation in the brain is age-dependent, having the lowest levels at birth and a marked increase with age [70]. Brain regions associated with motor function have a high concentration of iron [70], and its levels in the whole brain are around 35.6–54.2 µg/g [71]. Its accumulation in the brain affects neurons. Exposure of neurons to iron induces oxidative stress, causing lipid peroxidation and DNA damage, which leads to caspase-dependent apoptotic cell death [72]. 

Several key mediators of ferroptosis have previously been implicated in PD pathogenesis. The SNpc is an iron-rich, dopamine (DA)-producing midbrain nucleus, which probably explains why it has a high risk of suffering neuronal death [73,74], mainly when iron accumulates, representing a PD feature [27,75]. Iron produces hydroxyl radicals with subsequent dopamine oxidation, likely contributing to an oxidative environment that increases the loss of nigral dopaminergic neurons in PD patients [76]. Moreover, genetic disorders that result in brain iron dyshomeostasis often cause Parkinsonism [77,78,79], demonstrating increased iron’s potential to contribute to PD pathogenesis. Indeed, mutations in several proteins involved in iron transport, increasing iron uptake and decreasing its export, are linked to PD. Mutant forms of transferrin, a critical protein for neuronal iron uptake, are associated with increased susceptibility to PD [80,81]. These data suggest that the iron uptake mechanism is overactive in these patients resulting in increased neuronal iron accumulation.

Conversely, mutations in transferrin receptor 2 (TfR2) [81] are associated with a protective effect in PD, potentially due to reduced iron uptake. Neuronal iron export occurs via a transmembrane ion channel, ferroportin [82], and the Alzheimer’s disease (AD)-implicated amyloid precursor protein (APP) stabilizes ferroportin expression on the membrane to promote iron efflux [83]. In contrast, loss of APP membrane function results in impaired iron efflux and consequent neuronal iron retention [84]. Indeed, several rare variants of APP predispose individuals to PD, and several studies of familial AD indicate APP mutations are associated with Parkinsonism and LB formation [85,86,87,88]. Deficits in iron export in PD were further identified in the substantia nigra, with a significant depletion in APP expression levels independently of cell loss and an 80% decrease in ceruloplasmin (CP) activity [74,89]. Ceruloplasmin also has a ferrous oxidase activity and enables iron export by converting Fe^2+^ to Fe^3+^ [90], which is then bound to and removed by transferrin. Several point mutations in the CP-encoding gene are significantly associated with PD [91] and Parkinsonism [78,92], indicating that CP-mediated iron homeostasis is also likely involved in PD pathogenesis.

Vitamin C, or ascorbic acid, has been shown to improve the absorption of Levodopa in some PD patients with poor Levodopa bioavailability [93]. However, it might be toxic as it loses one electron and forms an ascorbate radical (Asc^•–^). The electron can reduce metal ions such as iron and copper. The acidic extracellular environment favors the reduction of protein-centered metal, represented as Fe^3+^ reduction to Fe^2+^. Subsequently, Fe^2+^ donates an electron to O_2_ forming O_2_^•–^ with subsequent dismutation to H_2_O_2_ [94]. Moreover, vitamin C administration may aggravate PD progression due to the possible peroxidation of Fe^2+^ bound to Asc^•–^. Therefore, combined vitamin C therapy for ROS scavenging and an iron chelator to sequester the metal may be a promising PD treatment option, reducing the toxicity induced by DA-derived quinones [95].

#### Current Status of Iron Chelation Therapeutic Effect on PD Patients

Iron chelation is a successful treatment for iron accumulation-based systemic pathologies, such as cardiomyopathy associated with hemochromatosis [96] and thalassemia [97]. Since iron accumulation in the brain has been linked to PD development, this metal chelation emerges as a promising therapeutic target [90,91,92].

Studies in animal PD models have shown that iron chelation reduces and stops the pathological accumulation of α-synuclein [98] and decreases oxidative stress [99,100] when administered focally, intranasally, and even orally. In addition, some sophisticated translational studies (Table 1) demonstrated that iron chelation therapy decreases labile iron and oxidative stress in vitro and in vivo, ending with a pilot clinical study that reported symptomatic improvement in PD patients [101].

These promising results justify using iron chelating agents in clinical trials. In these studies, deferiprone (DFP) doses of 20 mg/kg/day and 30 mg/kg/day were well-tolerated by patients. In addition, decreasing iron levels in the dentate and caudate nuclei were detected by MRI, with a consequent improvement in the Unified Parkinson Disease Rating Scale (UPDRS) scores [102]. These improvements were observed more markedly in patients with low CP activity [103]. However, these promising results were not reproduced when a more extensive study was carried out in patients without dopaminergic treatment, where the disability increased over 36 weeks, suggesting that iron accumulation is only an early temporary compensatory mechanism to increase dopamine synthesis; however, in the long term, it worsens cell death [104].

Despite this, the initial results are still promising. Nevertheless, doubt remains regarding the effect of long-term iron chelation at the systemic level, as it could affect the circulating white blood cell number and iron homeostasis in cerebral glial cells, which is essential for processes such as myelin production by oligodendrocytes [105,106]. This controversy confronts us with the challenge of finding ways to modulate iron, not affecting other cells. Interestingly, lactoferrin, a cationic iron-binding glycoprotein, can cross the blood–brain barrier through transferrin receptor 1-mediated transcytosis on the surface of the brain capillary endothelial cells [107]. Lactoferrin protected from dopaminergic neuronal loss in a PD model induced with the neurotoxins MPP^+^ (1-methyl-4-phenylpyridinium) /MPTP (1-methyl-4-phenyl-1,2,3,6-tetrahydropyridine) by upregulation of brain-derived neurotrophic factor (BDNF), hypoxia-inducible factor 1α (HIF-1α), along with extracellular regulated protein kinases (ERK) and cAMP response element-binding protein (CREB) activation, and decreased phosphorylation of c-Jun N-terminal kinase (JNK) and P38 kinase [108]. Moreover, pretreatment with human lactoferrin positively affected the nigrostriatal system recovery after acute exposure to MPTP [109]. These results propose a new strategy for the regulation of cerebral iron homeostasis.

**Table 1:** Summary of deferiprone iron chelation effect on PD clinical trials.

Study Design	Clinical Trial	Subjects Male:Female (m:f)	Outcomes	Reference
Randomized, double-blinded, placebo-controlled clinical trial	Phase 2	22 subjects: •8 placebo (m:f) 3:5 •7 DFP 20 mg/kg/day (m:f) 4:3 •7 DFP 30 mg/kg/day (m:f) 5:2	Brain iron chelation by DFP therapy was well-tolerated; there was an associated reduced dentate and caudate nucleus iron content with a trend for improvement in motor-UPDRS scores and quality of life, not statistical significance.	[102]
Randomized, placebo-controlled clinical trial	Phase 1	40 subjects: •21 early start DFP 30 mg/kg/day (m:f) 12:9 •19 delayed start DFP 30 mg/kg/day (m:f) 13:6	Most DFP-treated patients displayed clinical and radiological improvements. Those with lower CP activity appeared to respond better to iron chelation.	[103]
Randomized, double-blind, placebo-controlled, parallel-group, single-center trial	Phase 2	40 subjects: •21 early start DFP 30 mg/kg/day (m:f) 12:9 •19 delayed start DFP 30 mg/kg/day (m:f) 13:6	SN iron levels and UPDRS motor scores were reduced in patients with higher CP-ferroxidase activity in serum and CSF.	[101,110]
A multicentric, parallel-group, placebo-controlled, randomized clinical trial	Phase 2	372 subjects: •186 placebo •186 DFP 30 mg/kg/day	DFP without dopaminergic treatment worsened the handicap at the PD diagnosis time compared with placebo over 36 weeks. This finding provides evidence that the iron accumulation in the nigrostriatal pathway is a powerful short-term compensatory mechanism for increasing dopamine synthesis but possibly at the expense of long-term worsening iron-related cell death.	[104]

### 4.2. Copper

Copper is a trace element that constitutes 70 parts per million of the Earth’s crust. However, it is an essential micronutrient found in small amounts in tissues and cells, with a high concentration in the kidney, liver, and brain [111].

This metal functions as an essential cofactor and is required for structural and catalytic proprieties of more than 30 necessary enzymes; among them are ceruloplasmin, cytochrome oxidase, lysine oxidase, dopamine-hydroxylase, ascorbate oxidase, tyrosinase, and Cu/Zn SOD [112]. In living organisms, copper is mainly found oxidized (Cu^2+^) and reduced (Cu^+^) [113]. 

After the liver, the brain is the organ that accumulates the most significant amount of copper, reaching contents between 2.9 to 10 µg/g wet weight [114], and it is distributed differently in each region. The regions with the highest concentrations of copper are the substantia nigra, cerebellum, hippocampus, and hypothalamus [115]. Copper plays a crucial role in essential processes in CNS, such as brain development [116], antioxidant defense, synaptic transmission [117], and acting as an enzyme cofactor with oxidoreductase activity [112].

**Figure 4:**
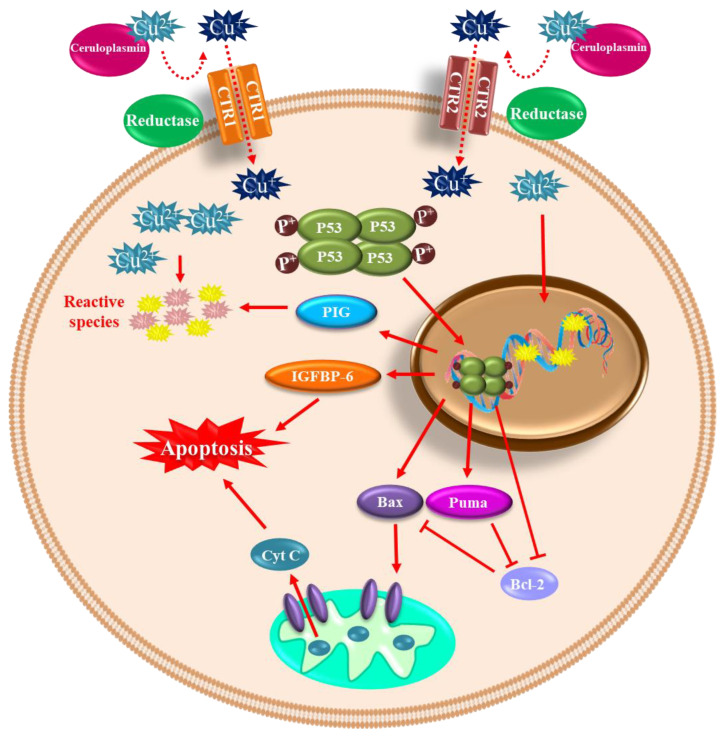
Copper-mediated neuronal cell death. Copper can be found in its oxidized (Cu^2+^) and reduced (Cu^+^) forms within the cells. The oxidized form of copper is bound to proteins, and when reduced by a reductase enzyme, it enters the cell through the transporters CTR1 and CTR2. Once inside and in high concentration, it induces DNA damage and p53 expression. p53 undergoes oligomerization/phosphorylation and is translocated into the nucleus to induce BAX and PUMA, with the consequent release of cytochrome C into the cytosol to initiate apoptosis. p53-dependent and independent apoptosis is triggered, where insulin-like growth factor binding protein-6 (IGFBP-6) and PIG proteins (prooxidant proteins), among other players, are involved.

Notwithstanding, like any redox-active metal, copper becomes toxic when its intracellular accumulation is excessive, facilitating the formation of ROS and apoptotic processes [118] (Figure 4). When the cell is exposed to oxidative stress or copper, DNA damage and p53 expression are induced [119,120]. Furthermore, p53 undergoes oligomerization and phosphorylation to be translocated into the nucleus to induce genes such as BAX (BCL2 Associated X) and PUMA (p53 upregulated modulator of apoptosis) and subsequent release of cytochrome C into the cytosol to initiate apoptosis [121,122]. Therefore, copper plays a vital role in many diseases, such as Menkes disease, where copper is abnormally low in the brain. Conversely, in Wilson’s disease, the damage is caused by an excess of copper stored in brain tissue. Moreover, some neurodegenerative disorders such as AD, amyotrophic lateral sclerosis (ALS), prion disease, and PD have been linked to copper dyshomeostasis [123]. Increased copper levels have been reported in the cerebrospinal fluid and blood of PD patients [124,125]. However, a recent meta-analysis reported decreased copper levels in the substantia nigra of PD patients compared to healthy age-matched subjects [126]. Nevertheless, it has been demonstrated that chronic occupational exposure to copper increases the risk of developing PD [127,128,129].

Copper toxicity affects the basal ganglia and frontal cortex inducing Parkinson-like symptoms and cognitive deficits. The mechanism of copper-mediated toxicity includes cell cycle arrest via the upregulation of p21 (Cyclin Dependent Kinase Inhibitor 1A), reprimo (involved in regulating p53-dependent G2 arrest of the cell cycle and coded by RPRM gene), stathmin (microtubule destabilizing protein coded by STMN1 gene), and Tp53INP1 (Tumor Protein P53 Inducible Nuclear Protein 1). Additionally, stat-3 (Signal Transducer and Activator of Transcription 3), hsp70 (Heat Shock Protein 70), and hsp27 (Heat Shock Protein 27) are increased in an attempt to survive. Finally, p53-dependent and independent apoptosis are triggered, where IGFBP-6 (insulin-like growth factor binding protein-6), glutathione peroxidase, BCL-2, RB-1, PUMA, and several members of the redox-active PIG family of proteins, play a role [130]. Moreover, copper binding to α-synuclein increases oxidative stress and α-synuclein phosphorylation and accelerates the protein aggregation process [34,131,132]. In the neuroblastoma cell line SK-N-SH with dopaminergic phenotype, copper transporter protein 1 (Ctr1) overexpression led to intracellular glutathione depletion and potentiated the caspase-3-dependent-cell death induced by copper, indicating that copper’s toxicity is due to alterations in its intracellular homeostasis. In addition, copper-induced oxidative stress was primarily localized in the cytosol, and Nrf2 was upregulated to mediate an antioxidant response. In addition, copper increased protein ubiquitination, AMPK-Ulk1 signaling, p62, and Atg5-dependent autophagy as a protective mechanism [133]. The release of redox-active copper ions from copper-binding proteins and its binding to thiol or amine groups of cysteinyl and histidinyl residues of globular proteins, including enzymes, may result in conformational changes leading to its inactivation [134,135].

Paradoxically, some clinical trials report decreased circulating copper levels in PD patients compared to healthy controls [136], which may occur, because by binding to ceruloplasmin, copper stimulates ferroxidase activity and participates in iron homeostasis. Therefore, low levels of copper can indirectly generate toxicity by altering iron concentrations [137].

Preclinical in vivo PD models showed that the chelation of heavy metals such as copper improved motor and non-motor deficits after MPTP intoxication [138], which has also been reproduced in other models of neurodegenerative diseases such as AD [139]. However, there is no evidence of any effects of copper chelators in PD patients in clinical trials. 

Since the strong chelation of metals can have systemic effects, alternative therapies targeting metal dyshomeostasis are critical. Recently, metal-protein attenuating compounds (MPACs) have emerged as promising therapeutic strategies. MPACs are moderate chelators that disrupt specific, abnormal metal-protein interactions [140] (Table 2). Under physiological conditions, MPACs bind to metal ions with a high affinity by competing with the metal-binding proteins to avoid their oligomerization and prevent the formation of metal-catalyzed ROS [141]. 

A novel ligand, 1-methyl-1H-imidazole-2-carboxaldehyde isonicotinoyl hydrazone (X1INH), attenuated abnormal copper^+^/copper^2+^-α-synuclein interactions and affected protein aggregation in a cellular model of synucleinopathy where inclusions were smaller and less compact [142]. Moreover, a moderate metal-binding compound, 8-hydroxyquinoline-2-carboxaldehyde isonicotinoyl hydrazine (INHHQ), was non-toxic to human neuroglioma H4 cells and was able to disrupt anomalous copper-α-synuclein interactions, probably by sequestering the metal ions. Importantly, INHHQ crosses the BBB and can be detected in rats’ brains as late as 24 h after its IP administration. After 48 h, brain clearance is complete, but INHHQ remains in the liver even 72 h after acute exposure. [143]. The effect of D-penicillamine, a relatively specific copper chelator, was assessed in the MPTP-induced PD mice model, showing a modest effect in preventing MPTP-induced striatal dopamine depletion [144,145]. In contrast, another study in the MPTP model detected a decrease in copper content in the striatum and midbrain, suggesting that its neurotoxicity is independent of copper [146]. Clioquinol (CQ, 5-chloro-7-iodo-8-quinolinol) can bind to the metal ions Fe^3+^, Cu^2+^, and Zn^2+^, which is why it plays a critical role in PD. Likewise, CQ remarkably improved the motor and non-motor deficits based on reduced iron content and ROS level in the SN [138]. HPCIH, HPCFur (pyridine-2-carboxaldehyde isonicotinoyl hydrazone, pyridine-2-car-boxaldehyde 2-furoyl hydrazone) has the ability to bind to Cu^2+^, which is why it has been linked to neurodegenerative diseases derived from misfolded prion proteins. HPCFur has a protective effect on methionine and histidine oxidation, which is related to physiological and pathological aging [147]. Therefore, physiopathologically relevant PD models reproducing the disease as in humans are urgent as the current models may not reproduce all characteristics of the disease and may lead to it through different mechanisms.

**Table 2:** Summary of metal-protein attenuating compounds (MPACs) effects on neurodegenerative diseases.

Metal-Protein Attenuating Compound	Metal Ions Binding	Neurodegenerative Disease	Outcomes	Reference
X1INH 1-methyl-1H-imidazole-2-carboxaldehyde isonicotinoyl hydrazone	Cu^+^ Cu^2+^	Parkinson’s disease	X1INH increased the number of smaller, less compact inclusions in a well-established model of α-Syn aggregation.	[142]
INHHQ 8-hydroxyquinoline-2-carboxaldehyde isonicotinoyl hydrazone INHHQ	Cu^2+^ Zn^2+^	Alzheimer’s disease, Parkinson’s disease	INHHQ can disrupt, in vitro, anomalous copper-α-Syn interactions through a mechanism probably involving metal ions sequestering.	[141]
Clioquinol (CQ) 5-chloro-7-iodo-8-quinolinol	Fe^3+^, Cu^2+^ Zn^2+^	Parkinson’s disease	CQ remarkably improved the motor and non-motor deficits based on reduced iron content and ROS level in the SN.	[138]
HPCIH, HPCFur pyridine-2-carboxaldehyde isonicotinoyl hydrazone pyridine-2-car-boxaldehyde 2-furoyl hydrazone	Cu^2+^	Misfolded prion protein	HPCFur has a protective effect on methionine and histidine oxidation, which is related to physiological and pathological aging.	[147]

## 5. Concluding Remarks

Nearly 40% of our proteins need a biometal as a cofactor to fulfill their function. Therefore, it is crucial to understand the transition metals’ role in health and disease because their dyshomeostasis (deficiency or overloading) is closely related to different disorders and mainly to neurodegeneration. Research in this field has found that biometals are tightly regulated because a narrow unbalance provokes diseases such as PD. Therefore, understanding the complexity of the interaction between transition metals and proteins can shed light on possible neurodegeneration biomarkers for preventing neuronal cell death. Hopefully, neurodegeneration may be achieved by either supplementing transition metals when there is a deficiency or using chelating agents to avoid metal overload that induces neuronal cell death in PD. 

The lack of success of metal-chelating agents in PD clinical trials is partly due to the lack of suitable models for its preclinical testing. So far, there is no animal model combining chronic exposure to metal ions emulating environmental and occupational exposure and aging, which may reflect how humans develop PD, as it is a multifactor disorder. PD animal models, like many others, are short-term, which has enormously contributed to our understanding of the mechanism implicated. However, it is time to combine the aging process with other risk factors, including environmental and occupational exposure, to develop more accurate PD animal models so that their translation into clinical trials leads to a higher probability of success. Why have we not used long-term models? The answer is simple; the main limitation is the time, followed by the increased resources required to feed, treat, and take care of mice. However, this approach may be fundamental to improving our chances of success in clinical trials.

## Data Availability

Not applicable.
